# Environmental factors of obesity before and after COVID-19 pandemic: a review

**DOI:** 10.3389/fpubh.2023.1213033

**Published:** 2023-12-18

**Authors:** Irena Anna Wolińska, Krzysztof Kraik, Rafał Poręba, Paweł Gać, Małgorzata Poręba

**Affiliations:** ^1^Divison of Pathophysiology, Department of Physiology and Pathophysiology, Wroclaw Medical University, Wrocław, Poland; ^2^Students’ Scientific Association of Cardiovascular Diseases Prevention, Wroclaw Medical University, Wrocław, Poland; ^3^Department of Internal Medicine, Occupational Diseases and Hypertension, Wroclaw Medical University, Wrocław, Poland; ^4^Division of Environmental Health and Occupational Medicine, Department of Population Health, Wroclaw Medical University, Wrocław, Poland; ^5^Department of Paralympic Sport, Wroclaw University of Health and Sport Sciences, Wrocław, Poland

**Keywords:** obesity, overweight, COVID-19 pandemic, environmental factors, eating habits, pollution

## Abstract

In past decades the prevalence of overweight and obesity had grown rapidly. There are numerous factors contributing to this unfavorable change in people’s health. This review article investigates the environmental factors which may play a role in the prevalence of overweight and obesity and additionally the novel factors which appeared after the beginning of the COVID-19 pandemic, which caused the increase in BMI during the lockdown period. Most of the studies reveal that the COVID-19 pandemic and lockdown contributed to the growth of BMI in numerous countries and, eventually the prevalence of overweight and obesity increased. Studies suggest that the physical activity was decreased while sleep time and screen time were increased and the amount of food consumed increased, additionally more processed food with long shelf life was consumed. The diverse environmental factors may have an impact on obesity and overweight development taking into account policy and local school policy issues, socioeconomic status, lifestyle including physical activity, diet habits, and amongst others, more trivial causes such as uninteresting neighborhoods, lack of sense of security outside the place of residence or a long distance from shops. Still, this is the object of debate if air pollution is an environmental risk factor influencing the unfavorable trends towards increasing body weight.

## Introduction

Nowadays, overweight and obesity are serious healthcare problems in most countries. The prevalence of overweight and obesity has been increasing globally continuously for several decades (1) and it seems that this trend will not change soon. Overweight is usually recognized when the BMI of an adult person is in the range of 25.0–29.9 while obesity is recognized when BMI is equal to 30 or it is higher. It is worth noting that the localization of adipose tissue is often overlooked in statistics. Central obesity, also known as visceral obesity may be in such cases neglected and, because of that, metabolically obese normal-weight people are not included in statistics, so the real prevalence of obesity might be higher. Also in some Asian countries the norms of weight BMI should be lower than it is accepted in Western countries (1, 2). Normal BMI in Asian populations is accepted as 18.0–22.9, the overweight range is 23–24.9 and obesity is when BMI is equal to or higher than 25 (2).

**Figure 1:**
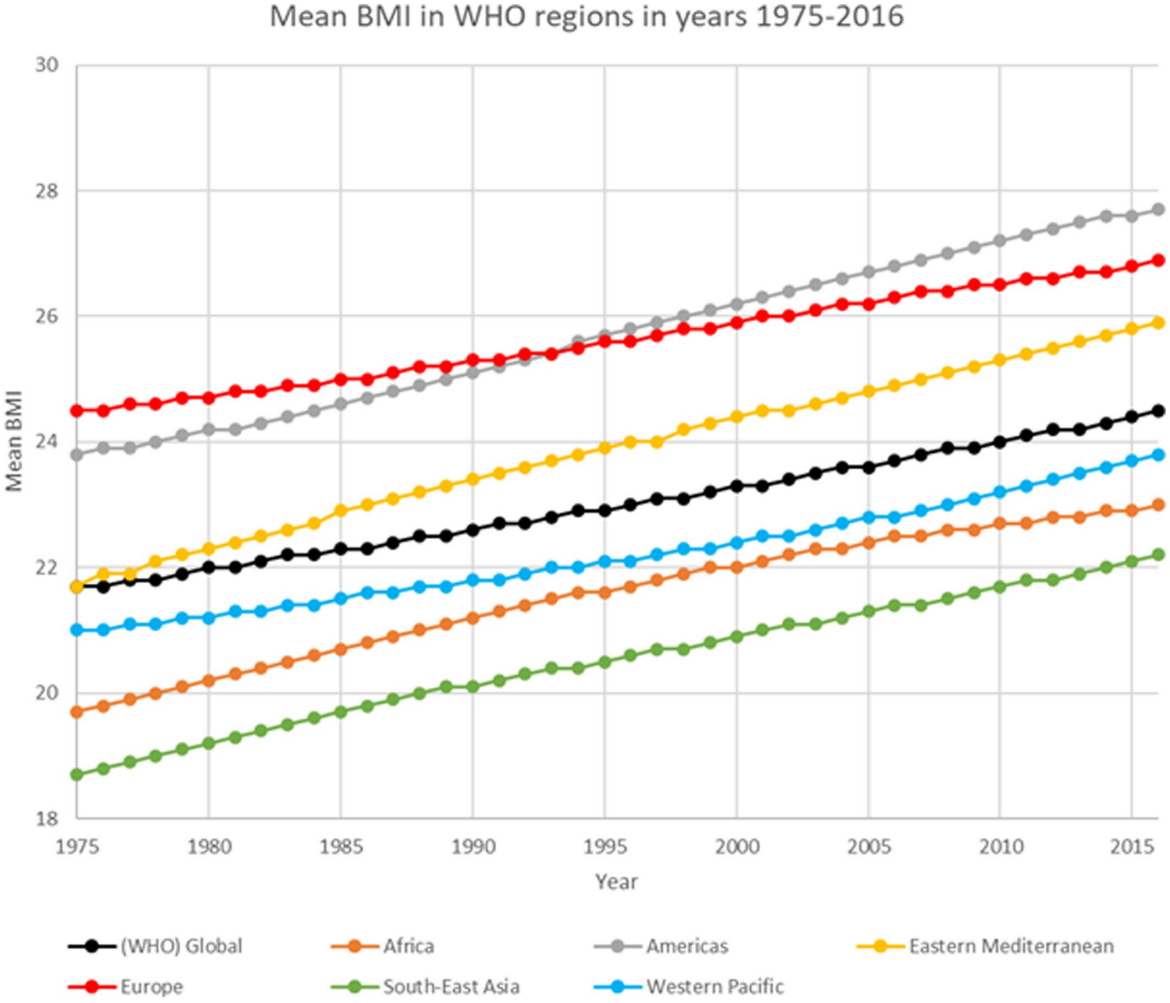
Mean BMI in WHO regions based on WHO’s Global Health Observatory data (5).

Prior to the COVID pandemic nearly 1 in 3 people worldwide was classified as overweight or obese (1). The number of people with too high body weight was rising rapidly. The prevalence of overweight and obesity doubled in the years 1980–2015. The rise in prevalence was most intense in the years 1992–2002. Obesity was more frequently affecting women and older people. In wealthy countries it affected mostly people with low socioeconomic status while in poor countries it affected mostly middle-aged people living in wealthy urban environments (3). Before the age of 45 women are less often obese than men but after that age women were more often obese than men. It might be linked to menopause. The main causes of obesity were identified as diet, lifestyle and socioeconomic status (4). The significant changes in mean adults’ BMI in different regions of the world in the years from 1975 to 2016 according to WHO’s Global Health Observatory (5) are presented in Figure 1.

Numerous factors may increase the risk of development of overweight and obesity including genetic, environmental, behavioral, biological, social and psychogenic ones (2). Among those, the most significant factors are physical activity, alcohol consumption and socioeconomic status. Moreover, the interplay of genes and environment further increases the risk of the development of overweight or obesity (6).

In late 2019 the SARS-CoV-2 virus responsible for the disease called COVID-19 was recognized in China. It spread rapidly to other countries and became a danger worldwide. As a result, on the day 11 March 2020, COVID-19 was declared a pandemic by WHO. To slow down the rate at which the virus spreads the governments of many countries declared a lockdown and encouraged people to stay at home and to keep a social distance. In most countries the lockdown had been expected to last a few weeks only, but then it was extended several times, which, eventually, enforced a change in people’s habits. It affected various daily routines including eating behaviors and physical activity. In addition, remote education was introduced in public schools and remote work became more common. It is possible that this change of behaviors affected people’s weight all over the world and, as a result, it could also contribute to the increase in the prevalence of overweight and obesity, which are conditions related to a higher risk of cardiovascular diseases, cancers and diabetes mellitus – the leading death causes worldwide (7, 8). Furthermore, both overweight and obesity also contribute to a more severe course of other diseases including COVID-19. Several studies confirmed that most of the patients admitted to Intensive Care Units in the pandemic era were overweight or obese and it had been found that both conditions increase the risk of respiratory failure in COVID-19 patients (9).

The goal of this study is to investigate the up-to-date knowledge on environmental risk factors of obesity and overweight, especially considering the influence of the COVID-19 pandemic.

## Methods

The non-systematic literature review was conducted using the following databases: PubMed, Cochrane Library, Embase and Google Scholar. Articles published between 1st January 2003 and 30th June 2023 were included. Different types of articles were included: systematic reviews, meta-analyses, reviews, clinical trials, randomized controlled trials, books and documents. A special focus was placed on systematic reviews and meta-analyses published since 2018 as these articles contain up-to-date information and they have the highest level of evidence.

In the search conducted in the databases we used a combination of groups of phrases to find publications related to the subject investigated by us. The first group of phrases included: “overweight,” “obesity,” “body weight,” “weight gain” and “food consumption.” The second group of phrases included: “environmental factors,” “environment,” “risk factors,” “epidemiology,” “pandemic,” “COVID-19,” “SARS-CoV-2,” “lockdown,” “coronavirus,” “air pollution,” “water pollution,” “pollution,” “pollutants,” “smoking,” “e-cigarettes,” “work,” “shift work,” “night work,” “circadian rhythm,” “eating habits,” “transport,” “rural area,” “urban area,” “climate,” “global warming,” “daylight hours,” “depression,” and “stress.” We used the conjunction “AND” in databases search boxes to connect both groups of phrases. We connected one phrase from the first group and one or more phrases from the second group in a single search. The duplicates were removed.

In the next step articles’ titles and abstracts were screened to qualify them to full-text reading. The inclusion criteria were: (a) studies related to the investigated subject, (b) English or Polish language, (c) studies published in peer-reviewed journals, (d) human studies. The exclusion criteria were: (a) animal studies, (b) abstracts without full-text article, (c) conference proceedings. We made an exception to one study (10) investigating the effect of nanocolloids in drinking water on obesity in mice due to the lack of similar studies performed on the human population.

We obtained the full text of articles that initially met our criteria and during the full text read articles that not met all inclusion criteria or met any of the exclusion criteria in the full text were removed and finally 58 articles were included in this review. Types of articles and the number of articles of a given type included in this review are presented in Table 1.

**Table 1:** Qualitative list of articles included in the current review.

Type of the article	Number of articles of a given type
Systematic review	8
Meta-analysis	11
Original research	24
Review	13
Editorial	1
Comment	1

**Figure 2:**
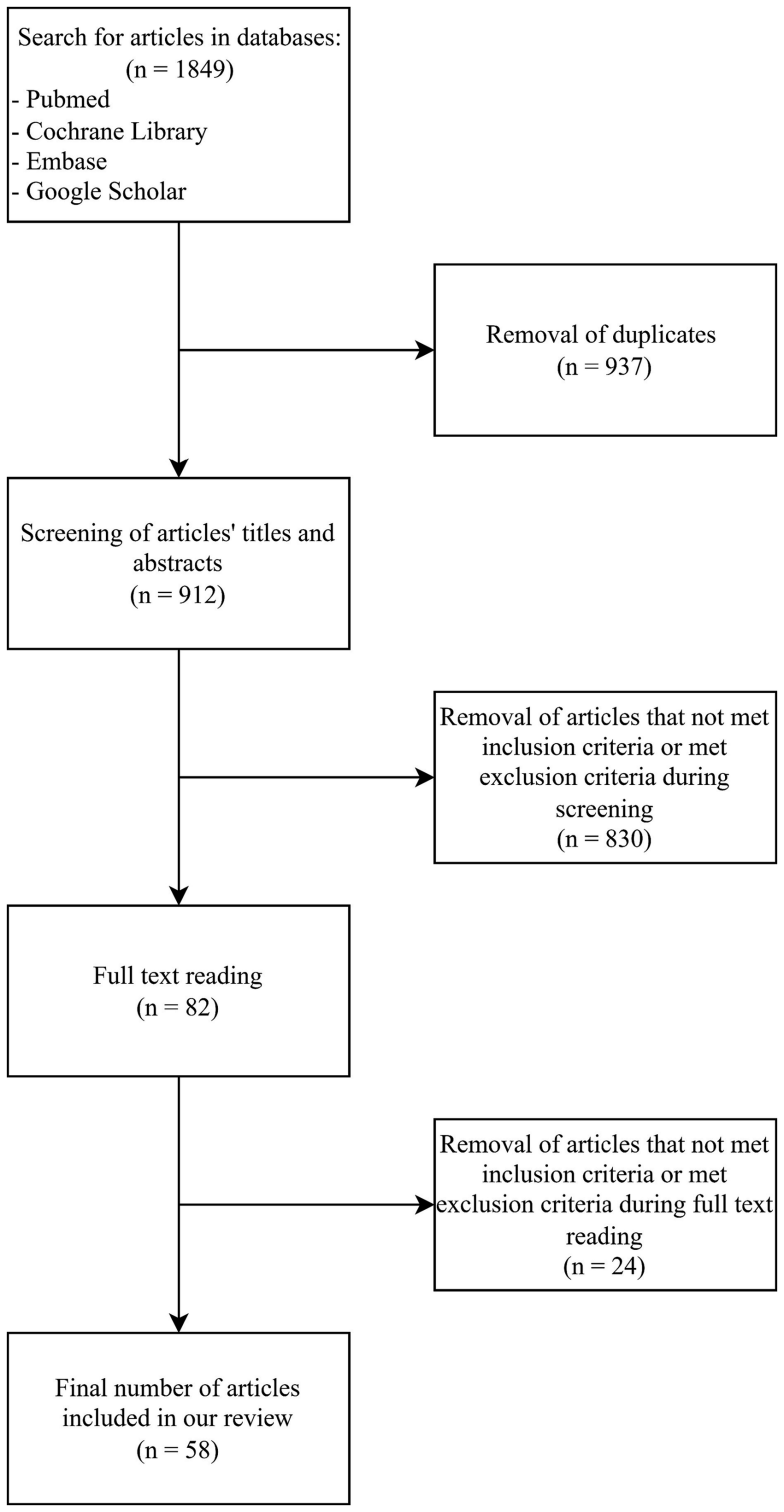
Summary of the article selection process.

The entire process of selection of articles was presented in Figure 2.

## Environmental risk factors of obesity and overweight

### General features

In the last century, various changes including the industrialization of food production (2) have been introduced, which made the world a more obesogenic place. There are some types of overweight and obesity risk factors. The prevalence of overweight and obesity is influenced by age, sex, race, and socioeconomic status (11). The environment in which people live has many components that increase the risk of developing these conditions. The environmental risk factors include geography, food availability, work environment and transport-related factors. The prevalence of obesity is higher in some regions than in others. Even regions of the same country may have different rates of obesity (12). In the United States the rate of obesity is higher in rural areas than in urban areas (12, 13), which may be an astonishing fact. However, the influencing factors include access to healthy foods and, paradoxically, fewer opportunities to be physically active (13). Moreover, it may be caused by differences in education level and income of residents of these areas and by the local infrastructure (11). Food availability is determined by how easily people can get a certain type of food. When healthy food is not easily accessible it might contribute to the growth of obesity prevalence (14). Difficult accessibility may be related to both prices and distance to the store being the source of food (11). It has been found that decreasing the distance to the shop by opening a new one in a nearer area positively affects people’s diet (15). Furthermore, when unhealthy food is easily accessible, for example in a nearby fast-food restaurant, it may also increase the risk of obesity development (16).

Advertising of fast food and calorie-rich food is another factor that increases caloric intake, and it especially affects children (17). Children are a vulnerable group that can be manipulated easily into buying certain products so many advertisements are aimed at them. Advertisements may shape their needs and preferences. It results in children buying products that have unfavorable effects on their health, eating more snacks and if that repeats, what is one of the aims of advertisements, they may carry harmful dietary habits to adult life or even develop overweight or obesity (18). Moreover, they may reduce the consumption of healthy food (19).

### Work environment

The development of technology caused changes in the work environment. Physical labor is less common than it was in the past and work-related screen time increased. Simultaneously, people do not need to expend that much energy during work time and eventually it is associated with increasing body weight (20).

The popularization of shift work was another change that increased the risk of developing overweight and obesity, especially abdominal obesity (2, 21, 22). The most adverse effect was observed in people working permanently on night shifts. Mechanisms proposed to explain body weight gain are circadian rhythm disorders associated with the inability to adapt to working at night and sleeping during the day and sleep deprivation (21, 22). Other mechanisms included more opportunities to eat during night shifts, hormonal disturbances and fatigue, which promotes eating more and reduces physical activity (22). Bonham et al.’s study found that the energy intake in the groups of shift workers and day workers was similar so the weight gain may be caused by meal timing, the type of consumed food and circadian rhythm disturbance (23).

### Natural vs. built environment and transport

The means of transport also affect the prevalence of obesity (11). In areas in which people are more willing to walk the overweight and obesity rates are lower (24). People prefer to walk in areas with a good landscape, which have good pedestrian infrastructure including sidewalks and paths and which have parks and recreational facilities (25). However, people are more reluctant to be physically active in places that are dangerous because of high crime rates (26) and traffic-related risks (27). Because of that people living in well-kept locations with extensive pedestrian infrastructure are less likely to be overweight and obese while people living in areas that are neglected or have high crime rates or huge traffic are more likely to develop obesity. It is also known that environmental factors interact with the individual factors of a person (11). A study conducted in Nigeria found that people living in developing countries in Africa are affected by similar overweight and obesity risk factors to those in developed countries. The neighborhood which was inviting to go out was linked to lower overweight and obesity rates while poor and dangerous areas were associated with higher overweight prevalence. The presence of garbage, unpleasant smell, crime and long distance to shops were factors linked to being overweight. There were also other factors that were significant but only to males or only to females. Lack of good pedestrian infrastructure and low residential density increased the overweight rate in males. Meanwhile, heavy traffic and the lack of interesting surroundings in the neighborhood were associated with higher overweight prevalence in females. Other factors that might contribute to the occurrence of overweight in developing countries are bad transport infrastructure, lower income and the status of being married. It was estimated that environmental factors increased the risk of being overweight by 40 to 60% (27).

### Leisure time

In other studies authors suggest that numerous other factors might be associated with the prevalence of obesity (28). The lack of recreational facilities may increase the chance of obesity in younger children by 68% (29). People who spend 3 h daily watching TV have two times greater prevalence of overweight than people who do not watch TV (28). These people are also more likely to be obese. Spending much time using smartphones and playing video games is even more likely to contribute to developing obesity because during these activities people often eat junk food which contains many obesogenic ingredients (28).

### Smoking and eating habits

It has been found that smoking before and during pregnancy increases two times the risk of developing obesity during childhood (28). Moreover, gaining weight after smoking cessation is a very common phenomenon (30, 31). The cause of gaining weight after quitting smoking is excessive calorie intake, decreased resting metabolism rate, decreased physical activity and increased lipoprotein lipase activity (32, 33). However, smoking is harmful to the extent that the health damage from weight gain is less than the damage from continued smoking (32). Fortunately, there are interventions to prevent or reduce body weight gain, e.g., using bupropion (33), modifying diet or exercising (34). The role of e-cigarettes in terms of body weight is still unclear and requires more research on the human population. The conclusions of the current research are contradictory (35, 36). Some studies found that people using e-cigarettes had a higher prevalence of obesity than the normal-weight population. However, no significant causal link was found between e-cigarettes and obesity (36, 37).

Eating faster (38) and huge portions (39) of food are also factors that might contribute to higher calorie intake occurrence of obesity. Consumption of sweet beverages both with sugar and artificial sweeteners also increases the risk of body weight gain (28, 40). Poverty which is linked to low income and low education level also contributes to increasing obesity prevalence (41, 42). Social norms, prices of different types of food and fashion may both increase or decrease the rate of obesity occurrence (28). Families in which parents are overweight or obese have greater chances of having overweight children (43). This relationship is independent of genetic factors (28).

### Climate, sun exposure, depression and stress

Changing climate and global warming also might be factors that increase the risk of obesity (44). There are reports that more energy is expended to digest colder food, and simultaneously it means, more calories are acquired by eating food at higher temperature than eating the same food at cold temperature. However, the potential effect of global warming on body weight is not large and is even less marked than the effect of owning a microwave (45).

The low number of daylight hours may also contribute to body weight gain by developing depression which increases the amount of food consumed by affected people (46). According to Luppino et al.’s meta-analysis depression increases the risk of developing obesity in both men and women due to hormonal changes (chronic activation of the hypothalamic–pituitary–adrenal axis), usage of antidepressants and lifestyle changes including the decreased amount of physical activity, switching to an unhealthy diet and eating an excessive amount of food when they feel bad (47). It is worth noting that obesity also may contribute to the development of depression. This reciprocal association has been found in many studies (47–49). However, according to Mannan et al.’s study, the risk of developing obesity due to depression is higher than the risk of developing depression due to obesity (48). Furthermore, Kanellopoulou et al.’s study found an association between depression and obesity in children (50).

Similarly, chronic stress also may contribute to excessive body weight gain. Nowadays, due to the constant rush and ambition, people are almost constantly exposed to chronic stress. Stress affects weight in many ways including overeating, eating calorie-rich food, decreasing the level of physical activity, decreasing the amount of sleep, disrupting intentional weight control, disrupting HPA axis, disrupting the reward center, changes in the gut microbiome and modifying the amount of synthesized regulatory peptides and hormones (neuropeptide Y, leptin, ghrelin). Furthermore, stigmatizing obese people increases the amount of stress they experience (51, 52). Moreover, some people may be more susceptible to stress due to individual factors such as the level of glucocorticosteroids and their sensitivity to glucocorticosteroids (53).

## The impact of pollution on the prevalence of overweight and obesity

Because of the industrial development of the world the natural environment is becoming more and more degraded. The exploitation of the environment leads to climate change and the emission of pollutants, which decrease the quality of the air, water and soil. All these factors may cause adverse effects on human health.

**Figure 3:**
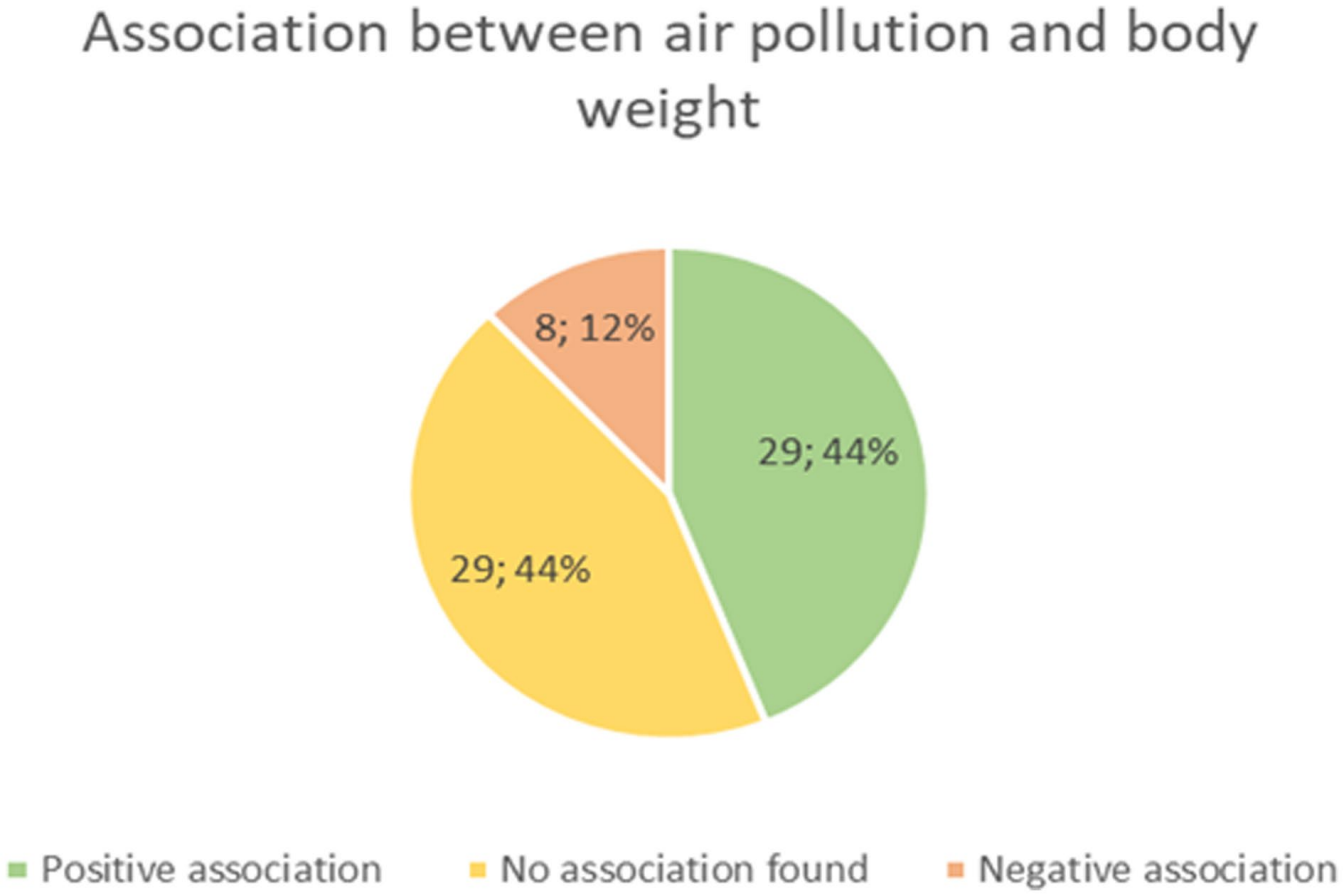
A number of studies which found positive, negative or no association between air pollution and body weight based on An et al. (54).

Air pollution is one of the most important environmental problems related to human health. Air pollutants are responsible for health problems including cardiovascular system diseases, neoplastic diseases and respiratory system diseases. These three types of diseases are the leading cause of death worldwide. The most important air pollutants are carbon monoxide (CO), lead, nitrogen oxides (NOx), ground-level ozone (O3), particulate matter (PM), and sulfur oxides (SOx). The impact of air pollution depends on sex, age and which pollutant is present in the air. There are some hypothetical mechanisms in which air pollution contributes to weight gain. The pollutants may cause oxidative stress and inflammations which leads to metabolic disorders. These metabolic disorders may further contribute to the development of obesity. Pollutants also contribute to other diseases like asthma which make people less capable of physical activities and as a result abstain from exercise. People are also less likely to go outside and exercise when they know that the air is polluted and when they see that there is smog. It also decreases the amount of physical activity. It is to some extent controversial if air pollution contributes to obesity, as a similar number of studies support or deny this idea. However, it should be highlighted that fewer studies have shown that air pollution contributes to decreased risk of obesity (54). Studies investigating the impact of air pollution on body mass changes according to An et al. are presented in Figure 3.

It appears that children are more susceptible to the obesogenic impact of air pollution than adults (54). An association between PM2.5 exposure and increased adult BMI was found while no association was observed when it comes to PM10 and NO2 exposure (55). It is likely that skipping activity due to pollution is the main mechanism by which air pollution affects the obesity rate in adults (54). It is also found that air pollution may slow down the metabolism of young adults and increase the risk of obesity (56). It has been found that even prenatal exposure to pollutants in the air may affect obesity (55). Children who were affected prenatally by polycyclic aromatic hydrocarbons (PAHs), NO2, PM2.5 or benzopyrene were more likely to be obese in their childhood. Smoking is a source of PAHs so children of women who smoke during pregnancies are more likely to be overweight or obese. Traffic air pollution may affect the metabolism of newborns and lead to obesity. Children living close to places with large traffic like major streets had higher BMI than those who live further to these places (55, 56). Another possible mechanism is affecting the endocrine system by pollutants (55). It is hypothesized that high levels of NOx from traffic may cause inflammatory changes. There are studies that found no associations between low-level NOx exposure and overweight or obesity rates in children. Most of the research point that air pollution increases obesity in children (54–56). The pollutants that are most significant in childhood obesity are PM2.5, PM10, and NO2 (56). PM may also cause sleep disorders that contribute to weight gain.

Water pollution also may affect the prevalence of overweight and obesity. Both organic and inorganic water pollutants may create the nanocolloids which are contributing to obesity. It was found that exposure to nanocolloids increased the weight of mice. After the exposure there was a change in gut microbes, namely toward the status that is commonly present in obese individuals. These microbes generate then long-chain fatty acids. Also, the level of leptin increased, and the expression of adiponectin decreased. Nanocolloids are also responsible for disorders in blood lipid metabolism (10).

## Overweight and obesity prevalence during the COVID-19 pandemic

### Children and youths

In 2020 the hypothesis was made that lockdown might affect children’s weight similarly to summer vacations because of the school closures (57). The hypothesis was based on the study which had been carried out prior to the pandemic. This study included a group of children whose BMI was monitored from kindergarten to second grade and it revealed that children’s BMI increased faster during summer vacation compared to the school year and that prevalence of overweight and obesity increased only during summer vacations (58), Table 2.

**Table 2:** A change of investigated parameters during school years and summer vacations based on von Hippel et al.’s study (58).

	Mean BMI	Prevalence of overweight	Prevalence of obesity
School years	↑	↓	↓
Summer vacations	↑↑↑	↑	↑

That suggested that school attendance might reduce the impact of risk factors causing the growth of BMI. The hypothesis assumed that the pandemic would increase the screen time and consumption of snacks and shelf-stable food, which is usually highly processed and less healthy, and that social distancing will reduce physical activity in children, especially those who live in an urban environment (57).

Low physical activity and high screen time are likely to be risk factors for overweight and obesity in children (59). The low sample (41 participants) longitudinal study which was carried out in Italy based on telephone interviews with parents of obese children supports this hypothesis. The food consumption during the lockdown increased in this group: the consumption of unhealthy food (red meat, potato chips, and sweet beverages) increased significantly. The consumption of fruits also increased but its significance is not marked as clearly as the increased consumption of unhealthy food. Also, the number of meals consumed every day increased, especially in the group of males. There was also observed a change in the amount of time spent on different activities: sleeping time and screen time increased while the amount of time spent on doing sports decreased (60).

Another large sample (10,082 participants) retrospective study carried out in China based on a social media survey supports the statement that lockdown contributed to weight gain in youths. The study included youths between the age of 16 and 28 years. The average age of participants was 19.8 years. The data about BMI, the prevalence of overweight (defined in that study as BMI ≥23) and obesity (defined as BMI ≥27) and the lifestyle of youths before and during the lockdown were collected. The mean BMI increased from 21.8 before the lockdown to about 22.6 during the lockdown. The prevalence of both overweight and obesity increased. The screen time and sleeping time increased. Most of the participants kept a moderate level of physical activity. However, the rest of them rather decreased their physical activity due to the lockdown. Also, the amount of time spent on transport-related actions like walking and cycling decreased during the lockdown (61).

Furthermore, the meta-analysis encompassing 12 studies (including the two mentioned before) revealed that children’s body weight and BMI have increased during the lockdown. Also, the prevalence of overweight and obesity increased in studied groups during the pandemic, especially in younger children aged from 5 to 9 years. The weight increase in the group of children affected by diabetes mellitus was not statistically significant. It was stated that the COVID-19 pandemic has worsened the epidemic of childhood obesity (62).

### Adults

The lockdown and COVID-19 caused unfavorable changes in adults as well. Both the COVID-19 pandemic and all the rules introduced by different countries to prevent infection like lockdown or social distancing caused the change in people’s diet and activity forms. In many cases the amount of physical activity decreased while sleep time and screen time increased. Additionally, the amount of food consumed increased. People were eating more processed food with long shelf life. Because of that it was more difficult for people to control their body weight. These changes likely contributed to body weight gain (62–66).

The study conducted in the UK revealed that due to the lockdown adults encountered many barriers which were hindering them from maintaining the proper body weight. There were 2002 participants who completed the questionnaire about their behaviors during the lockdown. Adults have eaten more snacks, especially the ones who had high BMI prior to the lockdown. People who had high BMI also were overeating more frequently and had worse diet quality than people with normal BMI. The diet quality of most participants worsened. Because of panic people bought a lot of highly processed food with long shelf lives and ate it instead of fresh, nutritious and healthy food which was less accessible during the lockdown. Levels of physical activity were also lowered, again especially in the group of people with higher BMI as many people were afraid to exercise outside. Because of that people lost control of keeping their weight within the correct values (63).

In the meta-analysis including adults, weight gain was observed in 12.8–29.9% of cases during the lockdown (64). In one of the Iraqi studies the weight increased in over 30% of people (65). On the contrary, according to Italian authors weight loss was observed in 35.7% of people while weight gain was observed only in 11.1% of people, although, the study group included only people aged 60 and more (66). In another study including only obese people 36.3% of them gained weight during lockdown (67). The lockdown had the greatest impact on those who were already overweight and obese (63). Moreover, it was also found that younger participants gained weight faster than older ones (64).

Most likely lockdown contributed to the acceleration of weight gain, growth of BMI and increased prevalence of both overweight and obesity. This fact is particularly unfavorable because excess body weight is one of the factors associated with the severe course of COVID-19 (9).

## Conclusion

Most of the studies reveal that the COVID-19 pandemic and lockdown contributed to the growth of BMI in many countries and in different populations of people and it increased the prevalence of overweight and obesity. Restrictions introduced to prevent infection including lockdown and social distancing caused changes in people’s diet and activity forms. The physical activity was decreased while sleep time and screen time were increased and the amount of food consumed increased. More processed food with long shelf life was consumed. However, still it should be remembered that the background of the development of obesity and overweight is complex, and it employs a variety of components such as socioeconomic problems, diet habits, lifestyle, type of work, and even the neighborhood view quality. Additionally, air pollution may be associated with obesity prevalence, especially in children, while the impact of water pollution on obesity is less studied.

## Author contributions

IW and MP: conceptualization. IW and KK: resources and writing—original draft preparation. PG, RP, and MP: writing—review and editing. IW and KK: visualization. RP and MP: supervision. PG: funding acquisition. All authors have read and agreed to the published version of the manuscript.
